# Concurrent cerebral arterial and venous sinus thrombosis revealing celiac disease- a case report and literature review

**DOI:** 10.1186/s12876-020-01483-w

**Published:** 2020-10-06

**Authors:** Dalia Alhosain, Lamia Kouba

**Affiliations:** 1 Division of Gastroenterology and Hepatology, Damascus University Hospitals, Damascus University, Damascus, Syria; 2 Faculty of Medicine, Damascus University, Damascus, Syria

**Keywords:** Celiac disease, Stroke, Venous sinus thrombosis, Arterial thrombosis, Case report

## Abstract

**Background:**

Celiac disease is an autoimmune condition characterized by an inappropriate immune reaction against gluten. It classically presents as chronic diarrhea, bloating, and nausea in addition to malabsorption symptoms such as weight loss and micronutrient deficiency. We report the first case of coinciding cerebral infarction and venous sinus thrombosis unveiling the diagnosis of celiac disease.

**Case presentation:**

A 40-year old female patient with a four-day history of severe diarrhea presented with right hemiplegia and altered mental status. Imaging revealed left middle cerebral artery occlusion and left transverse and sigmoid venous sinus thrombosis, along with left jugular vein thrombosis. Her laboratory evaluation was notable for profound iron deficiency anemia, thrombocytosis, and hyperhomocysteinemia. Her positive anti-tissue transglutaminase IgA antibodies and ensuing duodenal biopsy confirmed the diagnosis of celiac disease.

**Conclusions:**

Celiac disease has a wide range of intestinal and extraintestinal manifestations and can present with thrombotic events in young patients with iron deficiency and hyperhomocysteinemia.

## Background

Celiac disease (CD) is an autoimmune disease triggered by the exposure to gliadin; a protein component of gluten, in genetically susceptible individuals. The dysregulated immune response damages the intestinal mucosa in the duodenum and jejunum, leading to malabsorption and a constellation of extraintestinal manifestations.

The clinical presentation of CD typically comprises gastrointestinal symptoms such as recurrent diarrhea, weight loss, abdominal bloating, and nausea or vomiting [1]. However, CD is commonly asymptomatic and may initially present with extraintestinal manifestations or with systemic complications of malabsorption syndrome, rendering this disease a diagnostic challenge.

Celiac disease rarely manifests with thrombotic complications and when it does, the hepatic vessels are the most commonly involved [2].

In this report, we describe the first case of simultaneous arterial and venous thrombosis presenting as the initial manifestations of celiac disease. A summary of three proposed mechanisms of thrombosis in celiac disease is also described with the supporting evidence from the literature.

## Case presentation

A 40-year-old woman presented to our emergency department with deteriorating mental status. One day before, she suffered from a severe headache unresponsive to analgesics followed by right hemiparesis 4 h later. The patient’s family reported that they could not reach proper medical care until the next morning when the patient’s mental status was deteriorating. She also had a 4-day history of profuse greasy diarrhea. Her medication history is notable for combined oral contraceptives for 9 years and short-acting beta agonists for asthma. Collateral history from family revealed few episodes of loose stools along with a poor appetite and weight loss of 15 kg over the last 5 years. They denied a prior history of abnormal bleeding episodes or any thrombotic incidents in the form of deep venous thrombosis, pulmonary embolism or cerebrovascular event. The family also confirmed that the patient did not experience any previous neurological manifestations. The patient does not use tobacco, alcohol, or illicit drugs.

Her family history is unremarkable and concerning manifestations of atherosclerotic vascular disease was noncontributory.

**Fig. 1:**
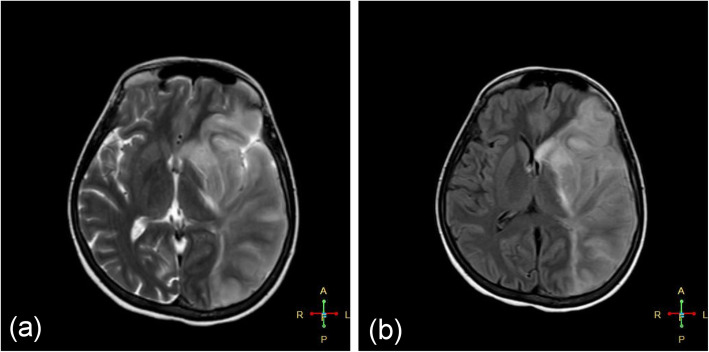
Axial MRI of the patient’s brain revealing a large hyper-intense area in the left parietal lobe corresponding to left middle cerebral artery (MCA) distribution (**a**; T2-weighted sequence, **b**; T2- weighted fluid-attenuated inversion recovery (FLAIR) sequence)

**Fig. 2:**
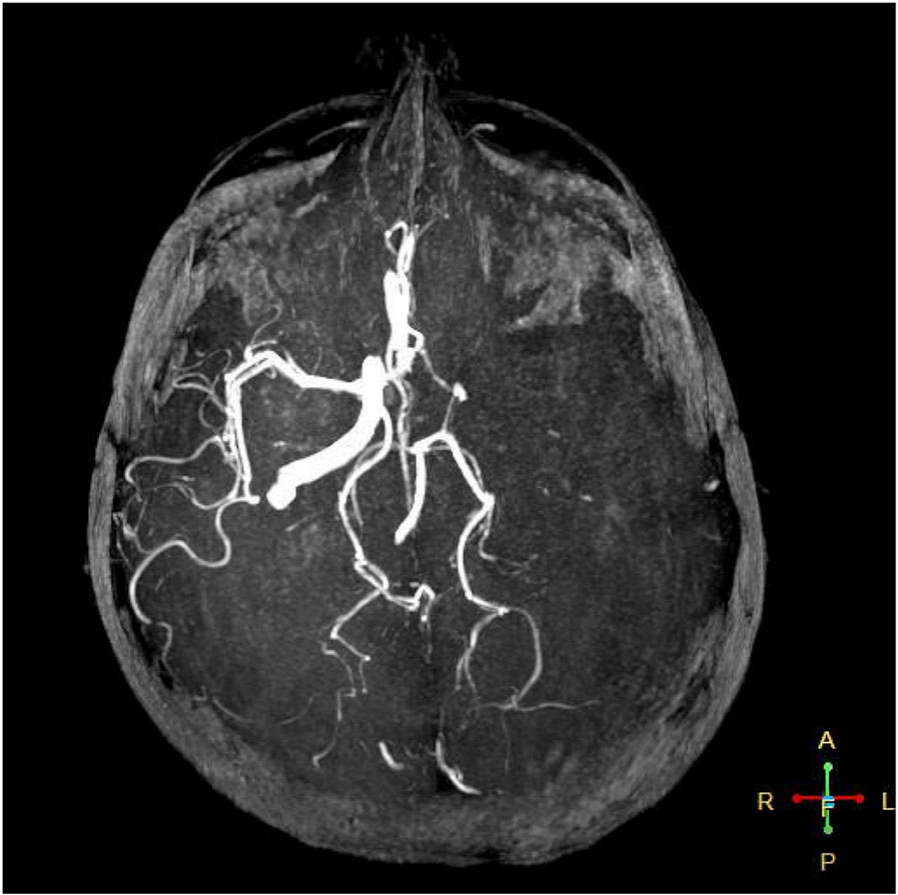
MRA of the patient’s brain revealing extensive left middle cerebral artery occlusion

**Fig. 3:**
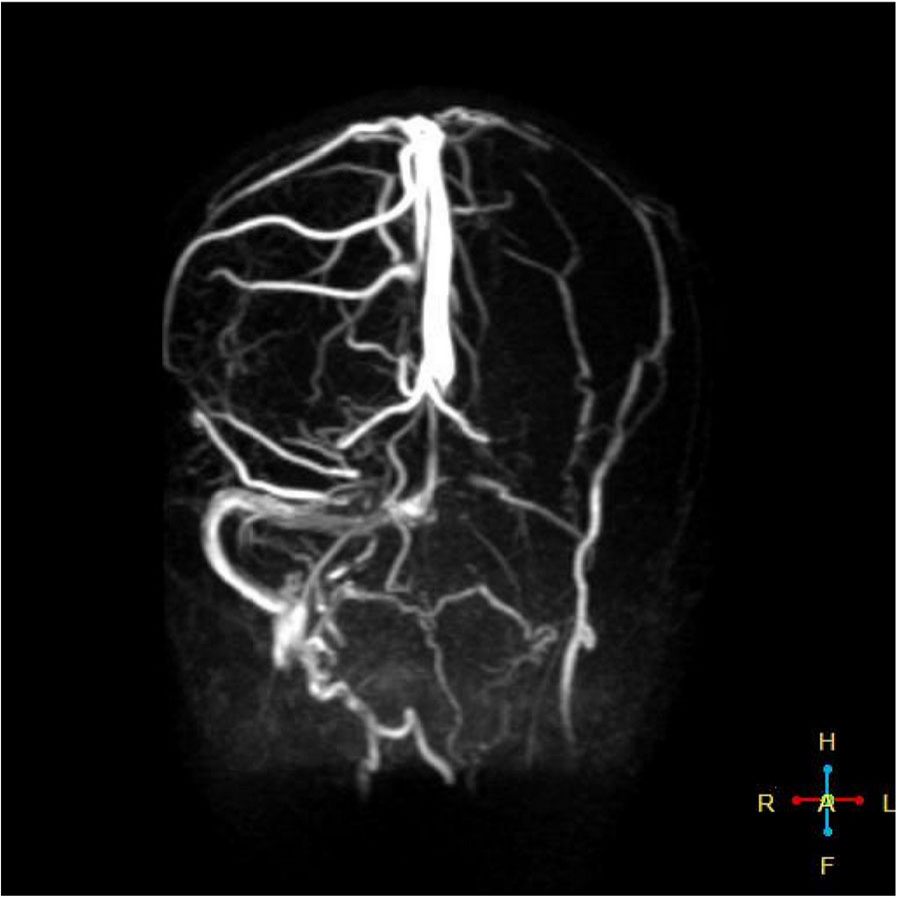
MRV of the patient’s brain depicting the thrombosis of the left transverse and sigmoid sinuses and the left jugular vein

Her initial Glasgow Coma Scale (GCS) score was nine. On physical examination, she appeared cachectic (BMI 17 kg/m^2^) and dehydrated. Marked weakness in the right upper and lower limbs was observed. The pupils were equal in size and response. A computerized tomography (CT) scan of the brain showed parenchymal hypodensity in the left parietal lobe. Magnetic resonance imaging (Fig. 1) and magnetic resonance angiogram MRA (Fig. 2) showed a large hyperintense area in the left cerebral hemisphere corresponding with left middle cerebral artery infarction. The magnetic resonance venogram (MRV) revealed left transverse and sigmoid sinuses thrombosis in addition to a thrombotic left jugular vein (Fig. 3). Duplex scanning of the neck vessels showed total occlusion of the left internal carotid artery and atherosclerotic right internal carotid artery with noncritical stenosis.

Laboratory evaluation revealed iron deficiency anemia with hyperhomocysteinemia and folate deficiency; Hemoglobin 80 g/L, Mean Corpuscular Volume 66 fL (80–95 fL), Ferritin < 1 μg/L (11–307 μg/L), Transferrin saturation 8% (20–50%), serum folate 2 nmol/L (4.5–45.3 nmol/L), homocysteine 15 μmol/L (5–12 μmol/L).

Serum potassium (K) and phosphorus (P) levels were low; K 3.2 mmol/L (3.5–5 mmol/L) and P 12 mg/L (26–45 mg/L).

Coagulation profile showed: INR 2, aPTT 28 s (30 s–40 s) and thrombocytosis with platelet count of 641 × 10^9^/L. Serum levels of protein S and C were low. Lupus anticoagulant screening was negative and anti-cardiolipin antibody test was also negative.

To investigate the etiology of the patient’s iron deficiency anemia, anti-tissue transglutaminase (tTG) IgA and IgG antibodies were assessed and found to be strongly positive: anti-tTG IgA 215 U/mL (positive: > 10.0 U/mL) and anti-tTG IgG 20 U/mL (positive: > 10.0 U/mL) and the total serum IgA level was within the reference range. Consequently, a duodenal biopsy was performed and showed marked villous atrophy, crypt hyperplasia, and lymphocytic infiltration within the lamina propria consistent with celiac sprue (Marsh III b). The above findings were compatible with the diagnosis of celiac disease complicated with secondary folic acid and iron deficiency, hyperhomocysteinemia, and proteins C and S deficiency.

The patient was promptly started on a high-caloric gluten-free diet via nasogastric tube and the diarrhea ceased within 2 days. Additionally, low molecular weight heparin for cerebral venous thrombosis was administered along with aspirin and a lipid-lowering agent for further arterial stroke prevention. Nutritional deficiencies were corrected timely.

The patient gradually regained consciousness during the first 2 weeks, she was able to follow commands and open her eyes spontaneously but she had expressive aphasia and right hemiplegia.

She was discharged 20 days after admission, her GCS score was 11. She received nursing care at home in addition to physical and speech therapy.

On follow-up 2 months later, the patient had no diarrheal episodes. She was able to walk with assistance and follow commands but still had expressive aphasia. The laboratory panel revealed an INR of 1 and electrolytes serum levels within normal limits. An improvement of hemoglobin level and thrombocytosis (Hemoglobin 150 g/L, platelet count of 360 × 109/L) was also noted.

## Discussion and conclusions

Celiac disease or gluten-sensitive enteropathy is an autoimmune reaction against gliadin, a protein component of gluten in genetically susceptible individuals. It is strongly associated with HLA-DQ2 and HLA-DQ8. Celiac disease is widespread globally for a prevalence of 1% of the world population. However, its prevalence varies widely according to the geographical location, age, and sex [3].

Celiac disease was defined by a set of classic standards for diagnosis. Nevertheless, the integration of serologic, genetic, and histologic data has led to the recognition of other varieties of celiac disease: the asymptomatic, latent, and atypical disease.

The classic definition of CD includes the presence of gastrointestinal manifestations such as bulky diarrhea or foul-smelling floating stools, often accompanied by malabsorption symptoms such as growth failure in children, weight loss, severe anemia, neurological symptoms, and osteopenia in adults. Additionally, the detection of duodenal villous atrophy on histology and the resolution of both the mucosal lesions and physical symptoms upon withdrawal of gluten-containing foods are characteristic features of the classic CD [4].

Asymptomatic (silent) celiac disease is usually discovered incidentally through screening tests for antibodies against tissue transglutaminase in asymptomatic people [5].

On the other hand, latent celiac disease describes CD patients who recovered completely with a gluten-free diet and remained asymptomatic even once a normal diet was resumed [6].

Patients with the atypical celiac disease lack classic symptoms of malabsorption but may exhibit minor gastrointestinal complaints. They usually have extraintestinal manifestations of celiac disease including anemia, osteoporosis, arthritis, chronically elevated transaminases, neurological symptoms, infertility, and several associated autoimmune diseases [7]. These patients usually have villous atrophy in duodenal biopsies and display positive celiac antibodies.

In our case, our patient had a history of weight loss, multiple episodes of loose stools, along with a poor appetite over the last 5 years. Her rapid mental status deterioration and one-sided weakness were the main symptoms that prompted her to seek medical treatment and eventually led to the diagnosis of celiac disease.

There are three main possible hypotheses concerning the pathogenesis of thrombosis in CD.

### Malabsorption-induced vitamin deficiency

The low levels of vitamin K result in proteins C and S deficiency, and the inadequate levels of vitamins B12 and B9 contribute to hyperhomocysteinemia.

Hyperhomocysteinemia has toxic effects on endothelial cells. Homocysteine enhances platelet aggregation, promotes endothelial factor V activation, and interferes with protein C activation and thrombomodulin expression [8]. Hence, hyperhomocysteinemia is recognized as an independent risk factor for vascular thrombotic disorders.

The deficiency of protein S or protein C results in the overactivity of coagulation factors V and VIII [9], thus increasing the risk for thrombotic events.

A brief description of published cases reporting such association in the literature is summarized in Table 1 [2, 8, 10–18].

**Table 1:** Case reports of thrombotic events in CD patients caused by malabsorption-induced hyperhomocysteinemia and Protein C and S deficiency

Year	Authors	Cause of thrombosis	Thrombotic complication	Number of cases	Patient demographics	Reference
2002	Gefel. et al	Hyperhomocysteienemia	Recurrent stroke, cardiac thrombosis coronary	1 case	Male, 33	[8]
2005	Bahloul. et al	Protein S deficiency	SVT	1 case	Female, 21	[10]
2006	McNeill. et al	Protein S and C deficiency	SMA thrombosis	1 case	Male, 40	[11]
2008	Audia. et al	Hyperhomocysteinemia, Anticardiolipine and anti B2 glycoprotein antibodies	Stroke MCA-M1	2 cases	Male, 43 and Female, 32	[12]
2009	Kallel. et al	Protein S deficiency	DVT leg	1 case	Male, 18	[13]
2009	Kochnar. et al	Protein S and C deficiency	Hepatic vein thrombosis	1 case	Male, 19	[14]
2010	Rachid. et al	Hyperhomocysteienemia	Stroke	1 case	Male, 52	[15]
2011	Berthoux. et al	Protein C and S deficiency, Hyperhomocysteinemia and anti B2 glycoprotein antibodies	Peripheral veins, descending aorta, portal and splenic veins	7 cases	Female (*n* = 6), Male (*n* = 1)	[16]
2013	Boucelama. et al	Hyperhomocysteinemia and Antiphospholipid antibodes, protein S deficiency	SVT	2 cases	Female (*n* = 2) 63, 19	[17]
2013	Ghannouchi. et al	Protein S and C deficiency	Intracardiac thrombosis	1 case	Male, 32	[2]
2016	Wassim. et al	Protein C and S deficiency	Retinal central vein	1 case	Female, 26	[18]

*SVT* Cerebral sinus venous thrombosis, *SMA* Superior mesenteric artery, *MCA* Middle cerebral artery, *DVT* Deep vein thrombosis

In three cases [8, 12, 15], hyperhomocysteinemia prompted an ischemic stroke before a diagnosis of CD was made, whereas in one case, hyperhomocysteinemia provoked cerebral venous thrombosis in a patient with untreated CD [17].

The first reported case describing protein S and C deficiency-induced thrombosis in CD was published by Ghannouchi et al. [2], where a patient had intracardiac thrombosis presenting with an ischemic stroke. Protein S deficiency is associated with other unusual locations of thrombosis, such as cerebral venous thrombosis [10, 17], the hepatic veins, deep veins of the lower extremities, and the superior mesenteric artery [11, 13, 14].

### Iron deficiency anemia with or without thrombocytosis

The second possible contributor to thrombotic events in CD is iron deficiency anemia (IDA) with or without concomitant thrombocytosis. However, its mechanism of action is still unknown. The reactive thrombocytosis secondary to iron deficiency is generally considered benign. However, it was shown that reactive thrombocytosis can cause prothrombotic states that may lead to severe and even fatal complications. A rare case of simultaneous thrombosis of a cerebral artery and cerebral venous sinus was reported by Ho et al. in a young female patient with iron deficiency anemia [19]. However, the patient had a uterine myoma causing her iron deficiency in addition to cryoglobulinemia and acquired protein C and S deficiency caused by previous therapy with warfarin.

Hartfield et al. reported a series of six children with ischemic strokes and venous thrombosis in whom iron deficiency anemia (6 of 6) and thrombocytosis (4 of 6) were the only positive laboratory findings in common [20].

Two cases of multiple peripheral and pulmonary thromboembolisms and cerebrovascular thrombosis were described in the literature, both cases were attributed to reactive thrombocytosis secondary to iron deficiency anemia [21]. A review of the published literature on thrombosis and thrombocytosis associated with iron deficiency anemia was conducted by Keung et al., 26 cases of cerebral venous thrombosis and ischemic infarcts (12 pediatric and 14 adult cases) were identified and the cause of IDA was reported [21].

### Central nervous system vasculitis associated with CD

The third incriminated factor contributing to a hypercoagulable state in CD is the presence of serum autoantibodies and central nervous system vasculitis in CD patients. Lerner and his colleagues conducted a study to investigate the thrombophilic complex of serum autoantibodies in celiac disease. They studied the two thrombogenic antibodies: antiphosphatidylserine/prothrombin (aPS/PT) and antiprothrombin. An increased incidence of antiphosphatidylserine/prothrombin aPS/PT IgG was detected in the CD group compared to the control group. Moreover, patients with CD were found to have higher activity rates for aPS/PT IgM and prothrombin IgG autoantibodies compared to the control group [22]. Therefore, thrombophilic serum autoantibodies are operative in CD and are a significant contributor to the hypercoagulability tendency of the disease.

In terms of autoimmune cerebral vasculitis, tissue transglutaminase is the main autoantigen responsible for maintaining the integrity of the endothelium. Therefore, the disruption of cerebrovascular transglutaminase could interfere with the integrity of the blood-brain barrier leading to the activation of autoimmune reactions within the central nervous system.

Pratesi and his colleagues demonstrated in vitro that anti-endomysial antibodies within the cerebral vasculature are present uniquely in CD patients on a gluten-containing diet [23].

The key finding of Korponay-Szabó et al. that anti-endomysial and anti-tissue transglutaminase antibodies are identical implies that the immunofluorescence observed by Pratesi et al. was the cerebrovascular transglutaminase [24].

The autoimmune reaction against transglutaminase in cerebral vascular endothelium can, therefore, be postulated as a cause for cerebrovascular accidents in patients with CD. To further support this hypothesis, Rush et al. reported biopsy-proven central nervous system vasculitis-induced stroke in association with celiac disease [25]. Furthermore, radiologic evidence of central nervous system vasculitis in a patient with recurrent stroke and celiac disease was also reported [27]. Published case reports of cerebral thrombosis in patients with CD attributed to autoimmune vasculitis are summarized in Table 2 [25–32].

**Table 2:** Case reports of cerebral thrombosis caused by CD-induced autoimmune vasculopathy

Year	Authors	Thrombotic complication	Number of cases	Patient demographics	Reference
1986	Rush.et al	Stroke ACA	1 case	Male, 51	[25]
2001	Ozge. et al	Stroke MCA	1 case	Female, 51	[26]
2003	Morello. et al	Multiple strokes	1 case	Female, 32	[27]
2004	Elmoutawakil. et al	Stroke PCA, MCA-M2	2 cases	Female (*n* = 2) 32, 39	[28]
2004	Goodwin. et al	Stroke MCA	1 case	Female, 3	[29]
2010	Dogan. et al	Stroke MCA-M2	1 case	Female, 8	[30]
2012	Fabbri. et al	Recurrent stroke PCA	1 case	Female, 26	[31]
2015	Poulin. et al	Stroke MCA, aortic mural thrombosis, and popliteal artery thrombosis	1 case	Female, 40	[32]

*ACA* Anterior cerebral artery, *MCA* Middle cerebral artery, *PCA* Posterior cerebral artery

Taking into account the patient’s origin, an alternative explanation for thrombosis in this patient was considered. Behçet disease is described to be linked to celiac disease and shares a number of characteristics with CD [33]. However, since our patient had no history of oral or genital ulcers and no history of uveitis or visual complaints, a diagnosis of Behçet disease remains unlikely.

In the present paper, we described a novel initial presentation of celiac disease; simultaneous cerebral arterial and venous sinus thrombosis in a young woman with no previous history of gastrointestinal symptoms or atherosclerosis risk factors.

In our case, the undiagnosed CD instigated a prothrombotic state by two mechanisms: A) the malabsorption-induced vitamin deficiency mechanism, subsequently causing low levels of protein C and S and increased levels of homocysteine, a well-known thrombogenic factor. B) iron-deficiency anemia caused by iron malabsorption and with concomitant thrombocytosis leading to a hypercoagulable state in an unknown mechanism.

A possible limitation of our study was that the thrombogenic antibodies aPS/PT IgG or IgM antibody levels were not assessed in the patient’s serum.

Although iron deficiency and vitamin K deficiency may be encountered frequently in the clinical practice, their presence in context of CD has played a synergistic role in the instigation of such a severe thrombotic complication.

To elaborate further, in a recently published review conducted by Fousekis et al. it was demonstrated that celiac disease is responsible for inducing a hypercoaguable state that could result in thromboembolic events [34].

Fousekis et al. studied several pathogenetic mechanisms of thromboembolism in celiac disease including nutritional deficiencies due to malabsorption, chronic inflammation, protein C and S deficiencies, and thrombocytosis, and these prothrombotic factors were present in our case.

This report adds to the growing body of literature on the diverse manifestations of celiac disease and extends our knowledge on the extraintestinal symptoms that could prompt the diagnosis of celiac disease. Early diagnosis and treatment improve the quality-of-life for celiac disease patients and may spare them various long-term or even fatal complications.

## Data Availability

Not applicable.

## Abbreviations

CD: Celiac disease
IDA: Iron deficiency anemia
CT: Computed tomography
AGA: Antigliadin antibodies
DGP: Deamidated gliadin peptide
GCS: Glasgow coma scale
BMI: Body mass index
tTG: Tissue transglutaminase
MRV: Magnetic resonance venogram
MRA: Magnetic resonance arteriogram
MRI: Magnetic resonance imaging
aPS/PT: Antiphosphatidylserine/prothrombin
SVT: Cerebral sinus venous thrombosis
SMA: Superior mesenteric artery
MCA: Middle cerebral artery
DVT: Deep vein thrombosis
ACA: Anterior cerebral artery
PCA: Posterior cerebral artery
